# Student Learning in an Interprofessional Peer Shadowing Activity Embedded in an IPE Curriculum: An Aotearoa New Zealand Ethnographic Case Study

**DOI:** 10.1177/23821205261448297

**Published:** 2026-05-03

**Authors:** Eileen McKinlay, Julia Myers, Linda Gulliver, Sonya Morgan

**Affiliations:** 1 Centre for Interprofessional Education, 2495 University of Otago, Dunedin, Aotearoa New Zealand; 2 Department of Medicine, 8494 University of Otago Wellington, Wellington, Aotearoa New Zealand

**Keywords:** interprofessional collaborative practice, interprofessional education, New Zealand, rural, peer shadowing, students, ethnographic observational methods

## Abstract

**Background:**

Shadowing is described as an interprofessional education (IPE) learning activity where a student of one discipline shadows a registered health professional or student of another discipline. To date, research examining the educational effectiveness of the shadowing learning activity has largely focused on students shadowing registered health professionals. This study explores if and what students learn, and the factors that influence their learning, when they shadow students from other disciplines.

**Methods:**

This ethnographic, case study observational research used indepth analysis to undertake a fine-grained examination of interprofessional learning gained through student’s peer shadowing of each other in a 5-week IPE programme in a rural setting.

**Results:**

Both the students who were shadowing and students who were being shadowed were observed to learn in the activity and this learning was multifaceted. It included learning about another discipline’s roles, skills and philosophies and the different training programmes, as well as personal and professional concerns and also wider aspects of health care. Peer teaching resulted in active and sometimes reciprocal learning and students were for the most part confident and enthusiastic.

**Conclusions:**

Peer shadowing involving senior students of different disciplines where interaction and social engagement occur, promotes meaningful and comprehensive interprofessional learning, including enhancing understanding of interprofessional collaboration as well as other aspects of professional and social practice. It is important for educators to prebrief the students about the goals of peer shadowing and advise them to prepare themselves for the learning activity, but once done, incorporating peer shadowing within IPE curricula provides an effective learning activity in which all students benefit.

The **Alllin4IPE study** is a four-country research collaboration between Sweden, Norway, Australia & New Zealand, and funded by the Swedish Research Council. The study seeks to explore the interprofessional learning that occurs in interprofessional education (IPE) placements and identify aspects across countries that are critical to IPE design and delivery. The research uses ethnographic observational methods and is underpinned by the Theory of Practice Architectures (TPA) which provides a lens to examine how practices are shaped by cultural-discursive, material-economic, and socio-political arrangements.

The research team are:

Sweden: Lindh Falk, A (PI)., Abrandt Dahlgren, M., Hopwood, N., and Landberg, K. Norway: Iversen, A., Norbye, B. Australia: Mansfield, K., Metusela, C.

New Zealand: McKinlay, E., Morgan., S., Myers, J and Gulliver, L.

An international research study on interprofessional health professions education, ALLin4IPE - Linköping University.

## Background

Shadowing is proposed as a promising interprofessional (IP) learning activity for health and social care students to acquire interprofessional competencies.^1^ Although not formally defined, pre-registration IP shadowing has been described as a student accompanying and observing the day-to-day work activities of either 1. a registered health or social care professional from another discipline,^2-10^ or 2. a student from another discipline.^11^ Most published literature includes instances of students shadowing registered health or social care professionals rather than students shadowing other students. In our own country of New Zealand there is one example of what is described as shadowing where junior doctors worked alongside registered nurses for two days and delivered nursing care.^12^

Published examples of IP shadowing involving registered health or social care professionals have described the shadowing activity as lasting from 2-10 hrs^1^ with one study of 56 hours duration^5^ and another of a week’s duration.^6^ The students’ stage or level of learning when shadowing is undertaken varies with year 2 or pre-clinical being the most commonly cited, suggesting shadowing is undertaken early to mid-way through a training programme. Some shadowing learning activities provide a structured template to support learning^6^ and others describe an adult-centred unstructured approach, where students are directed by what they themselves ‘notice’.^4,9,13^ The experience of shadowing is sometimes followed by a written reflection, oral presentation or an oral debrief.^1^

Shadowing other registered health and social care professionals helps students build competencies in IP collaborative practice.^1^ It does this by introducing students to the roles of other health and social care professionals, supporting them to develop an understanding of their own role in relation to other disciplines, and giving real-life experiences of IP leadership, IP communication and IP culture in the workplace. However, students shadowing other registered health and social care professionals has been critiqued as not meeting the interprofessional education (IPE) requirement to learn *with from and about*^14^ each other, as arguably the student is learning *from and about* but not *with* other students*.* In contrast, a student shadowing another student meets this requirement.

At the University of Otago, Aotearoa New Zealand, shadowing has been used as a student IP learning activity since 2012. We define it as: “a learning activity which gives students a chance to observe real-life examples of another discipline’s clinical practice (with patients or not), where the student/clinician of another discipline knows they are being observed while in authentic environments”.^15^ ^page 11^ This includes seeing and reflecting on IP communication, collaboration, shared decision-making, collaborative leadership and followership, and resolving difference. The learning outcomes for the shadowing learning activity are listed in Table 1.

**Table 1.** Learning Outcomes From the Shadowing Learning Activity

1. Explain your own health professional role and responsibilities
2. Recognise and value the roles and responsibility of other members of the health and social care team
3. Recognise when to consult with, refer to and/or collaborate with other members of the team
4. Demonstrate effective and respectful communications with other members of the team
5. Reflect on how interprofessional collaborative practice impacts on quality and effectiveness of care
6. Reflect on own perspectives and biases in relation to interprofessional learning and interprofessional collaborative practice

While evidence for the value of IP students shadowing registered health or social care professionals is growing, we can locate only one study where students of one discipline shadowed students of another discipline.^11^ This suggests a clear need to examine peer shadowing to determine if it is an effective IPE learning activity.

The overall objective of this ethnographic study was to examine peer shadowing within an IPE placement. Our specific aims were to identify:

• What and how students learn when undertaking peer shadowing activities with students from other health disciplines
• What influences students learning when undertaking peer shadowing activities with students from other health disciplines

## Methods

### Study Design

This study is part of a larger collaborative project, a 4-country multiple case study (the ALLin4IPE study) funded by the Swedish Research Council, which seeks to explore the IP learning occurring in IPE clinical placements, identifying aspects across countries that are critical to IPE design and delivery.^16^ The project uses ethnographic, case study observational research (CSOR) methods, theoretically underpinned by Practice Architectures theory,^17^ which provides a lens to examine how practices are shaped by cultural-discursive, material-economic, and social-political arrangements. The case study site for the project is a 5-week rural IPE clinical placement - the “Te Tai o Poutini IPE Programme” where students live together in shared accommodation and over 5 weeks undertake both uni-professional placements and a number of interactive IP learning activities (see Table 2 for a description of the IPE programme). The data reported in this paper focuses on student IP learning through the student peer shadowing learning activity, *one* of the of IP learning activities undertaken as part the Te Tai o Poutini IPE programme. The analytic approach taken in this paper is underpinned by a social constructivist theoretical approach, which posits that a person’s experience as a member of a group shapes their world view, and that students learn through social interactions.^18^ Ethics approval for data collection was granted by the University of Otago Ethics Committee (ref H23/045). The study is reported in accordance with the Consolidated Criteria for Reporting Qualitative Studies (COREQ).^19^

**Table 2.** The Te Tai o Poutini IPE Programme

The Te Tai o Poutini IPE programme is located in Greymouth, a remote, rural town established in the Gold Rush of the 1860s on the West Coast of the South Island of Aotearoa New Zealand (NZ). Greymouth has a population 8,600 and is a service town to a wide hinterland of farming, fishing, forestry and mining industries. In general, the needs of patients in the region reflect the demands of rural living and limited access to specialist care. Primary health care across the age span is offered through a small number of general practices including health promotion, illness prevention and acute and long-term condition care. Greymouth has a rural hospital which serves the whole of the West Coast (650 kms in length) and provides rural hospital medicine defined as a specialized, broad scope, generalist practice offering secondary care outside of urban settings.^20^ Accessing tertiary level care involves either a 3.5 hour drive over a mountain pass (often closed in winter time) to the nearest city (Christchurch) or a 30 minute drive to the closest airport (Hokitika) followed by a flight of at least 40 minutes duration. The IPE programme was established in 2021. Each rotation of the IPE programme is 5 weeks long with an intake of 6 to 12 students in each of the 5 rotations run during the year. The programme is funded by Health NZ (HNZ) Te Whatu Ora, the Crown agency which manages and plans NZ's public health services, while the University of Otago delivers the programme in accordance with the HNZ contract.
Students’ travel and accommodation are funded (no cost to students) and locally based and central academic and administrative staff are employed by University of Otago to run the programme. Students from 13 different disciplines and 7 tertiary training institutions can attend the programme, depending on availability (generally at least 4 different disciplines are in each block). Students have a mix of home discipline clinical placements (3 days per week) and interprofessional learning activities (2 days per week).^21^
The overall learning outcomes of the Te Tai o Poutini IPE programme are framed around the four key pillars of the programme: Hauora Māori (Māori health and wellbeing), Rurality, Long Term Condition Management and Interprofessionality. More generally learning outcomes for IP collaborative practice have been established by the University of Otago.^22,23^ As part of the 5 week programme, students participate in a range of different IP learning activities designed to support interprofessional competency development and knowledge of the rural community, including: a rapid real time community assessment, a community project, shadowing students from other disciplines, and interviewing as an IP group a patient with long term conditions. The scheduled learning activities target different types of learning, involve different numbers of students (small groups vs. all students), take place in different venues (clinical and non-clinical settings), and at various timepoints, intentionally staged over the course of the 5 weeks. In addition, there are many opportunities for informal interprofessional learning whilst students live together in shared accommodation.

### Participants

Two different rotations of the Te Tai o Poutini rural IPE Programme were selected for the case study research (March and July 2024). To meet the inclusion criteria, students were required to be in their final year of study in their health profession and thus be at an equivalent level of learning to other health professional students. Also included were shadowing placement supervisors with varying degrees of experience working with health professional students and experienced health professional clinical staff providing placements for students. Those excluded from the study were non-final year students and staff with no previous IPE experience.

**Figure 1.**
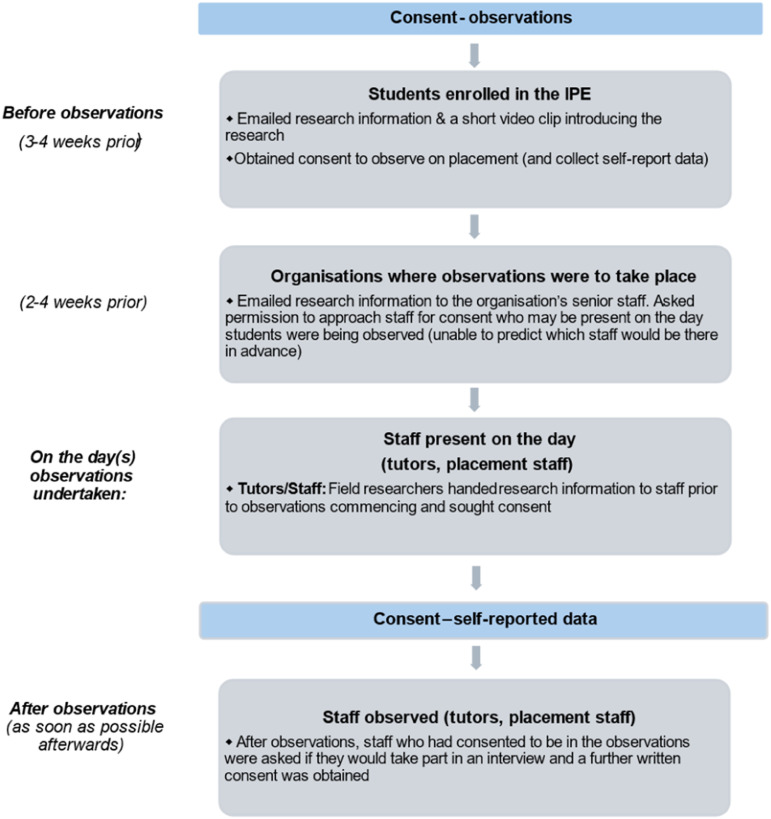
Process for the recruitment and consent of participants observed during shadowing placements. *Note.* Consent was also sought from patients present during the observations, but no data was collected from them. Placement supervisors not present during shadowing observations were separately approached for interview at another time

All students enrolled in each of the two rotations and who came from many different educational institutions and a variety of locations around NZ were invited to take part in the research by being emailed a study information sheet and consent form about 3-4 weeks prior to commencement of the IPE programme. They were also sent a link to a short video clip in which JM introduced the research and this was followed up by EM joining a routinely scheduled introductory Zoom video conference to answer any questions two weeks before each of the two rotations. Students were told the research would involve observation of their shadowing placements and the collection of other ethnographic data. The Information sheet gave the reasons for the research, the researchers interest and the topics to be covered in the interviews. Participants also included IPE placement supervisors/staff who were directly or indirectly involved in the student observations and/or participated in interviews. They were contacted by email several weeks before each rotation by SM along with being sent an Information sheet as above, and informed consent was gained from all parties prior to fieldwork commencing (see Figure 1). Students were informed the research did not involve any assessment of students’ individual performance and would not be used as part of their formal academic assessment in the IPE programme. No students declined to take part in the study. One IPE placement supervisor declined to participate due to unavailability and nominated another colleague for the research.

A total of 18 students (9 students in the first rotation and 9 students in the second) took part in the study, contributing observations and/or self-report ethnographic data pertaining to the shadowing learning activity. Students were from training programmes of varying lengths (3- 5 years).

A further 10 shadowing placement supervisors (indirect oversight) or staff (direct oversight) participants took part in observations and/or interviews (5 in the first rotation, 5 in the second). A description of participants is shown in Table 3.

**Table 3.** Description of Participants (Both Rotations)

Participants	
**Students**	
Discipline	Dental (4)
	Dietetic (1)
	Medicine (1)
	Medical Laboratory Science (1)
	Nursing (4)
	Oral health (2)
	Occupational Therapy (1)
	Pharmacy (2)
	Physiotherapy (1)
	Social Work (1)
Gender	Female (11)
	Male (7)
Previous IPE experience	Yes (6)
	No (11)
	Unknown (1)
**Placement supervisors (indirect oversight)**	
Discipline	Pharmacy (2)
	Physiotherapy (1)
	Dietetics (1)
Experience working with students in the IPE programme (rated by the local IPE staff)	Very experienced (2)
	Little experience (2)
**Placement staff (direct oversight)**	
Discipline	Dental (1)
	Dietetics (1)
	Oral health (1)
	Pharmacy (2)
	Physiotherapy (1)
Experience working with students in the IPE programme (rated by the local IPE staff)	Very experienced (2)
	Moderate experience (1)
	Limited experience (3)

### Data Collection

Data collection was informed by the principles of focussed ethnography^24^ and CSOR.^25^ Ethnography provides in-depth understanding about social processes and cultures in real-life settings.^24^ A focussed ethnographic approach examining a small number of shadowing examples was chosen for the research because focusing on a limited number of instances can provide rich, nuanced, fine-grained insights about the research phenomenon and does not rely on data saturation.^26^ CSOR preferences collection and analysis of observation data, and was chosen because observation allows the researcher to see what is occurring rather than relying on what people say they do through self-reported data sources such as interviews.^16^ Thus, consistent with CSOR, nonparticipant observations (where researchers observe participants without interacting or participating in their activities) of students during shadowing were undertaken prior to (and informed the collection of) other self-reported forms of data collection.^27^

Data from the two different rotations of the IPE programme in 2024 were collected over three visits to the programme site by three female researchers (JM, LG, EM). The field researchers are health professionals by background (JM physiotherapist, LG & EM nurses) and each have worked as trained researchers and educators, including in IPE, for at least 15 years. For each rotation two of the three researchers, all who live elsewhere in NZ travelled for a day to get to the remote programme site and undertook the site visits/data collection; one site visit of 2-3 days was undertaken with the first rotation of students in week 2 of the programme (JM & LG), and two site visits of 3 days each were undertaken by JM and EM with a second rotation of students (weeks 2 and week 5). In total approximately 30 hours and 45 minutes of student observations were undertaken (including of three different clinical learning activities-one of which was shadowing), approximately 2 hours 20 mins of interviews were undertaken with students, and approximately 4 hours and 20 minutes of interviews with shadowing placement supervisors and staff.

During the observations of students shadowing which occurred in hospital, community based health services (dental and pharmacy practices), or patient’s homes (each lasting between 35 mins to 3 hours 30 minutes), field researchers positioned themselves as unobtrusively as possible, (in the workplace this included being within earshot of where staff went about their work) and hand-wrote their observations as unstructured field notes in chronological order/with timestamps. A small number of illustrative photographs were taken with permission. An observation template was used as a guide for what to observe and record (see Appendix 1).

Other self-report ethnographic data collected from students included: 1) brief informal one-off interviews undertaken where possible, in a quiet spot in their placement sites immediately after observations, to ask students for clarification or perceptions of shadowing and recorded as hand-written notes; 2) audio-recorded one-off (50-60 minute) focus groups (either online or in-person in the student learning centre) were immediately conducted after site visit data collection (week 2 in the first IPE rotation, week 5 in the second rotation). Focus group questions focused on students’ perceptions of if and how the IPE programme, and the shadowing learning activity in particular helped students learn about IP collaboration and teamwork. Students were also asked about their experiences of working/learning together interprofessionally. 3) Electronic course discussion board posts routinely completed by students in their accommodation as part of the programme where students were asked to reflect on their learning experiences; 4) A weekly survey instituted in the second IPE rotation administered every Friday of the 5 weeks in the learning centre. It included questions about students’ experiences of working with students of other disciplines over the week and their perceptions of the IP learning experienced.

Shadowing placement supervisors and staff were also one-off interviewed, privately in their workplaces (20-40 minutes) to gain their views on the learning students undertake through shadowing. Brief informal one-off interviews were undertaken in their placement sites immediately after observations, to ask placement supervisors and staff for clarification or perceptions of shadowing, and recorded as hand-written notes.

As is typical in CSOR methodology, transcribed interviews were not returned for verification to student’s, placement supervisors or staff as they are not the primary data source.

Five examples of IP student shadowing placements/learning activities were observed: three in the first IPE rotation and two in the second. Each shadowing learning activity took place in a different setting (including the hospital, patient’s home, private dentist, private oral health clinic, community pharmacy). In all but one of the observations (pharmacy shadowing), patient(s) were present at times through the shadowing. In all but one of the placements (dental shadowing), tutors or other practice staff were present. Table 4 summarises each of the shadowing interactions observed, as well as the multiple sources of self-reported data collected with students and shadowing placement supervisors/staff and local IPE staff (student’s weekly survey, discussion board posts and focus group interviews and interviews with shadowing placement supervisors/staff and local IPE staff).

**Table 4.** Summary of the Examples of Shadowing Interactions Observed and Self-Reported Data Collected

Participants (students)	Location & time length of the observation	Patient present Y/N	IPE placement staff present Y/N
Observation data:			
Example 1: Nurse student shadowing Dietetics student	Hospital ward	Y	Y
	45 mins		
Example 2: Dental student shadowing physio student	Hospital gym & patient home	Y	Y
	1 hr 30 mins		
Example 3: Nursing student shadowing oral health student	Private oral hygienist exam room in a private dental surgery	Y	Y (at times)
	35 mins		
Example 4; Social work student shadowing Pharmacy student	Community pharmacy	N	Y (staff only)
	3hrs 15 mins		
Example 5: Med lab student shadowing dental students (2)	Private dental surgery	Y	N
	3 hrs 30 mins		

Participants	Informal interviews or clarification questions during/postobservations	Focus group	Interview	Weekly survey	Discussion board posts
Additional multiple sources of self-report data:					
Students	2	2	​	1	2
Shadowing placement supervisors/staff*	2	​	8	​	​

*Not all supervisors/staff were interviewed and some were interviewed together.

### Data Analysis

**Figure 2.**
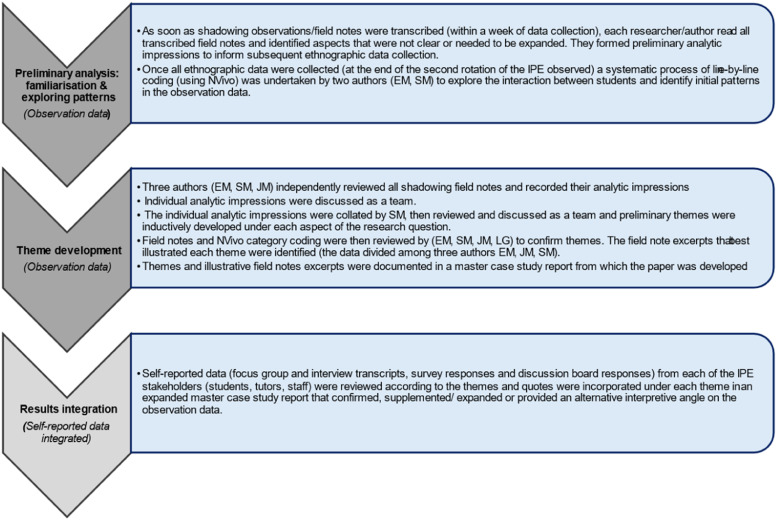
CSOR sequence of ethnographic data analysis

In accordance with the CSOR method, the analytic process was iterative, beginning with and prioritising the observation data throughout all stages.^25^ There were three main steps: 1. preliminary analysis of the observational data per example, 2. inductive theme development from the observational data which then included selection of illustrative data and 3. results integration which incorporated the other ethnographic data (see Figure 2). Students had completed their training programmes when the analysis was completed and were not able to provide formal feedback on the final themes although the themes aligned to the accounts they gave through the multiple forms of data collection.

## Results

**Figure 3.**
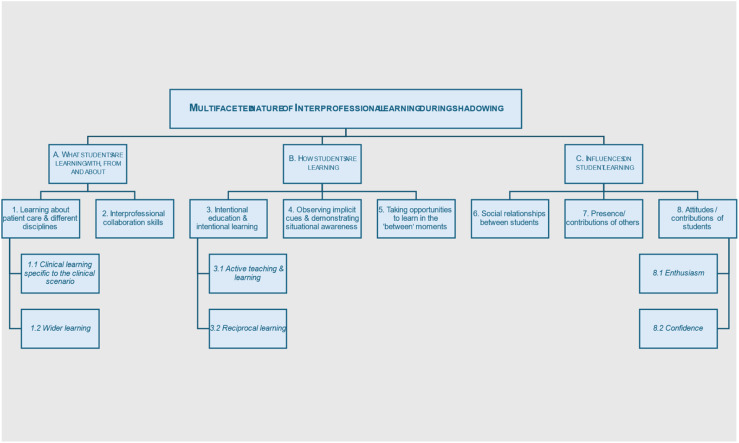
Themes and sub-themes

In-depth analysis of the five different examples of IPE peer shadowing showed a similar learning process occurring with variation in how students responded to it. Analysis identified the multi-faceted nature of students learning *with, from and about each other*. Themes and sub-themes were grouped under three major categories (see Figure 3 below).

A. **What** students are learning with, from and about
B. **How** students are learning in shadowing observations
C. **Influences** on student learning

For ease of reading the following abbreviation and symbols are used (see Table 5).

**Table 5.** Abbreviations and Symbols

Data sources
Electronic Discussion Board posts (EDBs), Fieldnote (FN), Focus Group (FG) Individual interview (II), Weekly Survey (WS).
**Disciplines**
Dental (Dent), Dietetics (Diet), Medical Laboratory Science (Med Lab Sci), Nursing (Nurs), Oral Health (OH) Pharmacist (Pharm) Physiotherapist (Physio) Social Worker (SW) There were two nurse students (1 & 2) and three dental students (1, 2, & 3)
**Abbreviations and symbols**
UTI (urinary tract infection) ECP (emergency contraception pill), COPD (Chronic Obstructive Pulmonary Disease), INR (International Normalized Ratio- a blood test used to monitor warfarin doses)
Brackets [ ] Used to identify whether the student is shadower or shadowee
Parentheses ( ) Used where the authors added words to clarify or replaced a student’s name with shadower/shadowee
Quotations appear in italicized font
“ ” direct quotes within fieldnotes

### A. **What** Students Are Learning *With, From* and *About*

During the shadowing placements, students were usually observed from the time they first interacted just prior to the shadowing formally starting until after they had debriefed at the end. The observations showed *what* students learnt and included content related to the broad clinical care of patients, professional roles and IP collaborative practice.

#### Theme 1: Learning About Patient Care and Different Disciplines

Although the shadower’s role in the learning activity was primarily to observe, in all but one of the shadowing placement examples, students were observed actively interacting with each other at various times during the shadowing, including before and after formal shadowing activities were complete. Shadowees (those being shadowed) were seen providing detailed explanations and/or demonstrating patient care/aspects of their professional role to the shadower (the student observing them).

Shadowers were observed to learn multiple things about the wider context of people who need healthcare and the clinicians who provide it, verbally articulating their understanding or using body language to signal learning (nods or other gestures and verbal affirmations). This was true for both learning specific to the particular clinical picture/scenario of the shadowed discipline and learning applicable beyond the specific/direct clinical observation/picture (e.g. education, social, service delivery, other aspects).

##### 1.1. Clinical Learning Specific to the Clinical Scenario

Observation of shadowees’ explanations/demonstrations and shadowers’ clarifying questions in each example indicated IP learning specific to the clinical scenario/activities being shadowed. Examples included shadowers being shown the professional equipment, and assessments and interventions specific to the discipline they were shadowing. It also included explanations or discussions between students about professional ethics, clinical reasoning and decision-making, rationing of healthcare resources, concerns/fears about learning, and how person-centered approaches occurred in the shadowed discipline.

The pharmacy student [shadowee] explains generic vs brand drug names. He makes the comment “there are lots of ways that pharmacists use to prevent error”. The social work student [shadower] asks a bit more about what kind of errors might occur and they talk about (medicines) dispensing machines and the machine in the shop that does blister packaging. The social work student affirms “all right, all right, yes I get that” and there's a lot of interchange between the two of them to check on understanding. (FN SW student shadowing a Pharm student)

However, the outcome of clinical learning was not always directly observable, as illustrated by the following example where there was no overt interaction between students or with the clinical tutor during the patient treatment.

(The nursing student [shadower] was observed to stand in the corner of the room… observing quietly, arms folded…peering at a distance over the clinical tutor’s shoulder to see what is going on with the oral hygiene clean (FN Nurs student 1 shadowing an OH student)

Although no visible interaction was observed, the nursing student subsequently self-reported by posting the following comment on the electronic discussion board, showing the learning, she had acquired through observing the oral health student.

When I was shadowing an oral health student, there was a patient that had a pacemaker. It was confirmed between the student, staff member and patient that the correct tools were used with this patient, to avoid their pacemaker being affected during their appointment. This showed that it is important for all relevant history of the patient to be disclosed prior to their appointment. It was also important for the student/staff to check each patient’s history and notes prior to the appointment, so these details can be confirmed with the patient, and everyone is on the same page. (EDB Nurs student 1)

Other students similarly noted that the shadowing experience had revealed clinical considerations they would incorporate in their own future professional practice.

(I learned) it is important to pay attention to patients’ general appearance and expressions, as well as stature to help build a comprehensive picture of any other conditions/problems the patient may be experiencing. This can help me better understand a patient's comfort and limitations when seeing the dentist. (EDB Dent student 1 shadowing Physio student)

##### 1.2. Wider Learning About Each Other’s Disciplines and Each Other as Health and Social Care Professionals

In addition to learning clinical information specifically related to the clinical scenario/activities being shadowed, students in the examples were also observed to learn more generally about wider aspects of each other’s disciplines. This included learning about each other’s disciplines’ philosophies, purpose, ways of being educated and focus of education, skills/capabilities, roles (and who they work with) and about the wider systems and context that the shadowed discipline worked within.

Students were observed openly sharing and learning about each other’s reasons for choosing their discipline, the road to qualifying, how skills are learnt in different disciplines, work opportunities, things they liked or didn’t like in their training, challenges in their discipline and so on.

As we all walked (through the hospital), the physio student [shadowee] and the physio tutor explained to the dental student [shadower] the different roles and types of things that physios actually do in hospitals [stroke rehabilitation, respiratory, etc.] (FN Dent 1 student shadowing a Physio student)

They started to talk about each other’s clinical placements and what sorts of things they do… Most of their communication was around their discipline-specific roles ….mostly the nursing student [shadower] was asking questions of the dietetics student [shadowee] about how she thought the visit might go and then more generally about their training. The dietetics student shadowee explains all their training requirements and the nursing student shadower asks clarifying questions. (FN Nurs student 2 shadowing Diet student- while waiting for the shadowing to begin)

[the pharmacy student] suggests they compound a cream which needs to be at a 3% strength when it normally is at 1% strength. … He says “Now this is how we do it”. … [the social work student says] “is this what you do at pharmacy school?” The pharmacy student says he practices a whole lot of different things at pharmacy school. (FN SW student shadowing a Pharm student)

They also shared their personal vulnerabilities in relation to learning particular clinical skills.

Some chit-chat between the two students … as the clinical tutor goes out of the room...Students are now sharing stories of how it can be scary in clinical learning and then having to demonstrate clinical skills. Shared feelings along the lines of needing to get assurance and to know that you are not hurting your patient. The oral hygiene student [shadowee] talked about doing a filling, and the nursing student [shadower] giving their first injection (FN Nurs student 1 shadowing an OH student)

Learning through shadowing extended to gaining insights about how the wider health system works.

While the students start talking about equipment … another staff physio enters the room and discusses the shortage of staff and whether they can cover a service. The physios’ conversation is not related to the student activity, but they are discussing equity versus outcome of going to provide a service for the patient who lives in an outlying community versus whether they could see more patients if they only visited the patients in the immediate area. The dental student [shadower] can hear some of this conversation from where he stands in the middle of the gym and expresses interest to the supervising physio… in how they organise services to find a balance between offering services to everybody and how they get the best outcome. (FN Dent 1 student shadowing a Physio student)

[the dental students- shadowees] then explain a little bit more about procedures and what they need to take into account in their role, for example they talk about financial cost and how they have to always explain this to the patient [prior to actually doing the treatment]. (FN Med Lab Sci student shadowing Dent students 2 & 3)

The observation that students were learning about the health system beyond the immediate clinical scenario, about each other and each others’ disciplines was confirmed by comments students later made in the survey.

…even if it's (shadowing a discipline) not directly applicable to your job…..(it) just gives you a broader perspective on how the how the health system actually works, and if you ever do give an opportunity to link in, with them (other disciplines), you understand who you're linking in with, who you're asking to help, … instead of just being kind of stuck in your own role, you don't really have a whole lot of perspective outside of it. (FG Nurs student 2)

#### Theme 2: Interprofessional Collaboration Skills - What Collaboration Looks Like

By working together during the shadowing placements, students appeared to be developing their own (or becoming more aware of/recognising) IP collaboration skills. This was demonstrated by numerous observations of both shadowees and shadowers in the various examples working to create a collaborative, supportive learning environment through behaviours such as providing encouragement/feedback, affirmations, clarifications, corrections, empathy or reassurance to each other, and supporting or helping each other.

The dental student [shadowee] tells the med lab sci student [shadower] to move a bit closer if she would like to see more, so she does move closer to see what's happening. Later the med lab sci student tells the two dental students that she was quite surprised how long it took (doing a filling) and how involved it was. “Gosh, you have to be quite artistic when you are doing this stuff. It's not just a question of fixing the teeth. You actually have to be very careful with things like getting the teeth even and matching the colours of the filling”… (FN Med Lab Sci student shadowing Dent students 2 & 3)

The use of genuine humour was observed to be used by students in different ways and functioned to support/strengthen collaborative working during shadowing. Humour could be used to facilitate a relaxed/safe practice and learning environment, defuse slightly awkward situations, or to express nervousness/unease, as well as providing reassurance.

The dental student [shadower] comments that he is already feeling a little bit lost in a big hospital because he's never been in one before. The physio student [shadowee] (laughingly) replies to him that “you actually get used to it really quickly”. (FN Dent student 1 shadowing a Physio student)

Observations reporting learning about IP collaboration during peer shadowing were confirmed by the self-report data. In a focus group at the end of the five-week block a student summarised their positive view of IP collaboration between students:

Yeah, … that's one of the things I've probably taken away (from shadowing) is that all of us as a group could look at one patient but we're all looking at different things for that one patient. (When we looked through the nurse's notes describing what the patient) had eaten, … (the notes) didn't actually go into the detail that a dietician would need to do their assessment of the patient. So yeah, seeing how that all plays together. (FG Nurs student 2 reporting her experience of shadowing dietetics student)

### B. **How** Students Are Learning in Shadowing Observations

The observation data also showed the process - *how* students learnt during shadowing observations, and included being actively intentional and preparing for the shadowing, being situationally aware and picking up on visual cues and using every opportunity (the in between moments) to share information.

#### Theme 3: Intentional Education and Intentional Learning

When students interacted during shadowing we observed in most examples that both teaching and learning was actively intentional and learning was sometimes reciprocal or bi-directional.

##### 3.1. Active Teaching and Learning

Many shadowees were observed investing considerable energy in actively teaching the shadowing student about their discipline, and using multiple educational techniques, suggesting they viewed their role as to educate (not just be observed). Field researchers noted that shadowees drew attention to things they thought would assist the shadower to ‘learn’ about the shadowee’s professional discipline and practice. This included aspects of practice outside what would be required to just provide direct clinical care and appeared to have been thought about in advance and specifically aimed at ‘teaching’ their fellow student.

(the pharmacy student [shadowee]) says “Shall I show you how to dispense a script so that you can see actually what I do”? He then shows the social work student [shadower] each part of the process. “So what we do is this”… After about 10 mins after all explanations are over, he prints off the label for the medicine and shows (the social work student) the machine that automatically dispenses and counts the pills. (FN SW student shadowing a Pharm student)

Shadowees appeared to intuitively know how to “teach” their fellow students and were often noted to be patient, kind and professional using behaviours they would typically use with patients. Some may have also learned this from their own experience of less successful shadowings.

During this conversation an assistant from the practice comes into the room to put some sterilised equipment away and [dental student 2- shadowee] takes this opportunity to explain to [med lab sci student - shadower] what all the different part bits of equipment she is putting away are for, and shows her different kinds of cleaning and other equipment, discussing the advantages of each. The med lab sci student asks about the next patient and (dental students 1 & 2) again take turns in explaining what is likely to happen. It's quite a comfortable and flowing conversation. Very interactive between the two dental students and the shadowing student. The dental students seem to be trying to think of things that might be relevant for (the med lab sci student) to know and then they explain them to her and show her all the various equipment associated with the potential problem that is coming in.. (FN Med Lab Sci student shadowing Dent students 2 & 3)

Similarly, shadowers were observed actively learning through asking questions or watching closely, and through verbal and non-verbal behaviours showing a genuine interest in the discipline they were shadowing. The example below shows active learning on both sides.

The pharmacy student [shadowee] offers to show the social work student [shadower] the consultation rooms and why they are used. “We can do a lot ourselves for UTIs, ECP” … “quite a lot of the stuff we do, we initiate ourselves not because GPs ask for it”… The pharmacy student says “lets look at some of the inhalers.” He shows the social work student the different types of inhalers and how they work. The social work student adds the capsules to the ones that use them and tries those with buttons etc. The social work student comments she has seen an inhaler before and it seems she is trying to build on this knowledge. (FN SW student shadowing a Pharm student)

The effort and reward of teaching another student was also described in a focus group at the end of the five weeks.

I think for me it [being shadowed] was quite stressful like initially like cos I just, yeah, I didn’t know how it was going to go and had never done it before … (But) it was actually a really cool experience. It’s kind of cool because it gets you to think about things that you do, but it’s like, why do you do it and sort of like…there is an art of being able to explain things to someone who hasn’t had the experience in that field. Yeah, I really enjoyed the experience. (FG Pharm student)

The intention by the shadowee student to make shadowing a rich learning experience for the shadower student was corroborated in the self-report data.

I know that when we had (the med lab sci student) shadow us … I certainly hope, we tried to be welcoming and engaging and you know. Like that wasn't quite the experience I had when I was doing the shadowing. (FG Dent student 3)

##### 3.2. Reciprocal Learning

Despite the pre-determined roles of shadowee and shadower, where the purpose was for the shadower to observe another discipline, the observations of the different examples showed the learning was not always a one-way exchange from shadowee to shadower. Although less frequent, shadowers were observed to contribute knowledge from their own discipline when the circumstances warranted it, and in return the shadowee could be observed taking an ‘active interest’ in what they were being told or shown by the shadower, hence demonstrating reciprocal or bi-directional learning.

Then there is conversation about mental health medicines. The social work student [shadower] has just been on a mental health placement where clients are on anti- psychotics and (she explains) they can't miss more than two doses otherwise medication has to be re-calibrated. The pharmacy student [shadowee] doesn't know about this so he's interested and asks more questions. (FN SW student shadowing a Pharm student)

The self-report data reiterated that the learning on both sides (shadowee and shadower) was mutual.

The shadowing student is very good. We share a lot of things with each other, giving more insight about (our different clinical work) (WS Dent student 2)

#### Theme 4: Observing Implicit Cues and Demonstrating Situational Awareness

In addition to the overt learning strategies displayed by students during shadowing, students in most examples were also observed to be engaged in more incidental learning. This was demonstrated by shadowers gathering information/implicit cues through close observation of their fellow shadowee student, and also the activities going on around them (i.e. reading the room). They demonstrated situational awareness showing insight or understanding into what was happening by responding to the anticipated needs of the situation (as opposed to learning from what was being explicitly communicated to them).

The patient doesn't appear very well and appears to be very thin and frail. After the patient has been speaking for a while, he starts to cough and neither the staff dietician or the dietician student [shadowee] can work out how to put the bed (head) up to stop his coughing, but at that point the student nurse [shadower] steps in and finds the controls for the bed and helps to put the bed up when she realises that the patient needs a drink. (FN Nurs 2 shadowing a Diet student)

Being situationally aware also meant important learning occurred by tuning in to incidental conversations between senior colleagues; in this situation a discussion about the need to trust other colleagues.

Two other physios in the room are still discussing their waiting list. The head physio seems to be advising the other physio about managing waiting list decisions He explains that it may be difficult for busy clinicians to provide all relevant details in a referral but you have to trust that even if all the detail isn’t there it is probable that the referral is still worthwhile otherwise the referring clinicians (usually a Dr or nurse) would not have gone to the trouble of writing it. The dental student [shadower] is listening attentively to this conversation, appearing to be taking it in. (FN Dent student 1 shadowing a Physio student)

Although less apparent in the self-report data, an example of situational awareness was noted where a shadowing student was aware when he recognised he needed to wait until he was in a staff-only area before asking questions.

I didn't really ask too many questions at the shadowing thing. It was more like afterwards or something when we were at a more in like a private area. [FG Dent student 1]

#### Theme 5: Taking Opportunities to Learn in the ‘Between’ Moments

Students were observed to readily take opportunities to interact and learn informally during (unplanned) ‘between’ moments of shadowing placements, outside of the immediate clinical interaction with patients or other clinical activities (for example whilst waiting for shadowing to commence/between seeing patients/walking between clinical areas/on tea breaks and during debriefs). This seemed to happen organically and appeared to be friendly and personable with no awkward gaps. These informal ‘in-between’ interactions most commonly related to learning about each other/each other’s disciplines more broadly - sharing/discussing work or clinical placement experiences, sharing information about their training or talking informally about living together, plans for the future including work and personal life, activities coming up in the evening and weekend. Sometimes students were observed using these ‘between’ moments to debrief about the clinical interactions they had experienced in shadowing.

The dental student [shadowee] asks the physio student [shadower] while they're waiting (to visit the patient) about how they learn how to use equipment and what type of lectures they learn it in. (FN Dent student 1 shadowing a Physio student)

*S*tudents self-reported that the shadowing experience in itself led to opportunities for, or primed, the shadowing student’s extra learning.

Before (I) arrived (at the clinical workplace), I was already asking (the shadowee physiotherapy student) some stuff. (FG Dental student 1)

### C. **Influences** on Student Learning

Finally, in the examples the observation data revealed three main themes *influencing* students’ learning during the shadowing observations. These influences on learning arose from students developing social relationships, the impact staff or others had on the shadowing process, and the attitudes and approaches of the students.

#### Theme 6: Social Relationships Between Students

Students were observed to socially interact with each other in a familiar way, beyond what might be expected to see amongst regular colleagues without an existing relationship. This more social relationship developed between students over time and could have arisen through a combination of attending a Noho marae (sleeping overnight together in a traditional Māori meeting house^28^) on the first day of the rotation, as well as living and working together for the 1-2 weeks prior to shadowing. Regardless, there was a free exchange of information evident in their interactions which appeared to facilitate student learning in the shadowing placements. Students were observed communicating openly together, sitting closely together, sharing personal experiences, frequently using humour, watching out for each other, and demonstrating kindness and empathy towards each other. They were also observed to be open about struggles/fears or challenges, or not knowing something.

(The shadowee dental students 2 & 3) are explaining to (the med lab sci shadower student) what the patient list is going to be for the day. They are telling [the shadower student] (about) each patient for the day and (what is likely to be required). … (shadowee dental student 2) asks (the shadower student) if she has observed any dentistry work before and comments, “well, you certainly have lovely teeth” (and then they all laugh together) and (the shadower student) responds “oh, do I, thank you”. … (Shadowee dental students 2 & 3) are both very open with communicating and seem to be making quite a big effort to explain everything to [the shadower student]. (FN Med Lab Sci student shadowing two Dental students 2 & 3)

This social closeness was affirmed in the self-report data.

…collaborating is a lot easier when it's with your friends or with people you know, or you've built some sort of like rapport with. And so, by having that socialising, getting to know people, the collaboration seems to be a bit smoother. It's not like awkward, I guess or, like you already know those roles and even as in a social setting, the roles often kind of transferred into the collaboration and the workspace. So I think that was… beneficial. (FG OT student)

#### Theme 7: Presence/Contributions of Others

Although other people were not always in attendance in the different examples, the presence (and contribution) of others (e.g. tutors) during shadowing was noted to influence the interactions observed. For instance, tutors had varying levels of experience, and although they had been given some instruction about what to do as a shadowing tutor, their understanding may have varied, potentially impacting the students’ experiences. Alternatively, tutors may not have been regular hosts of IP students. During two of the three shadowing placements where tutors were present, specific contributions made by the tutor appeared to facilitate the learning. These experienced tutors were observed to work skillfully to foster IP learning, not only for their ‘own’ discipline’s student but also the shadower student of another discipline.

The staff dietician overtly draws the nursing student [shadower] into their conversation, trying to include her. For example, she asks the nursing student what medications on the list of the patient that she recognises as a nurse, and the three of them discuss what the different medications might be for, and the nursing student is encouraged to explain to the dietetics student [shadowee] what the various medications might all be for. (FN Nurs student 2 shadowing the Diet student).

In one example, a student self-reported what they had learned from a tutor from another discipline.

(This) pharmacist is able to (directly) consult patients … like she can do a whole consult, she can take blood pressures, she can do, like the (extended) ‘capacity’ of what she can do, and she told me a few things I wasn’t aware of. … (she will check) the script from the hospital rather than (sending) them directly to the pharmacy. (FG Diet student)

#### Theme 8: Attitudes/Contributions of Students

Individual student factors –that appeared to be related to individual personality types-were also observed in the examples as shaping the way student interaction/learning unfolded during the shadowing placements. In particular, their personal enthusiasm and/or confidence.

##### 8.1. Enthusiasm

Students’ enthusiasm towards the shadowing activity was notable when they were observed to actively ‘buy in’ to the shadowing. This was particularly obvious in the two shadowers with skill sets which were either non-clinical (social work student) or clinical but not patient-facing (the med lab sci student).

[The shadowing med lab sci student], looks very interested and engaged… and the dental students [shadowees] are both very open. The med lab sci student is very engaged and tries to ask them questions. (FN Med Lab Sci student shadowing two Dental students 2 & 3)

The shadowees who were overtly enthusiastic appeared to be focused on showcasing their discipline. They showed pride in what their discipline did and were invested in their role of enabling their shadower to learn with and from them about ‘what they do’. Those who showed enthusiasm proactively provided additional explanations or demonstrated their skills before, during or after providing patient care and encouraged the shadower student to try it themselves, to help the shadower ’understand’ the shadowee’s clinical role.

The student physio [shadowee] visits the equipment cupboard to obtain a wheeled frame and a non-wheeled walking frame which may be needed for the home visit. Then the two students go to another cupboard to get a quad stick. The physio student is explaining to the dental student [shadower] what the different options might be and why you might need to try all of these different pieces of equipment out. … (The student physio) starts showing the dental student how a walking frame works. The dental student asks questions like the difference between the types of frames with wheels and without and what the different issues might be, when they are used. The dental student is trying out different types of equipment. (FN Dent student 1 shadowing Physio student)

The mutual benefit of an enthusiastic approach was also evident in the final focus group self-report data from a shadowee.

Just being able (as a shadowee) to explain what we're doing and it's a bit of reinforcement for us, but also you know a good way for us to, a good chance for us to explain what we're doing in clear terms to somebody who's never necessarily seen what we're doing. (FG Dent student 1)

##### 8.2. Confidence

Some shadowee students, more than others, appeared more confident/forthcoming or appeared to have undertaken a lot of preparation to facilitate the learning process of the shadower. They were overtly and actively trying to ‘teach’ about their discipline.

(The shadowee pharmacy student) then explains the other types of tests that pharmacists can do like blood sugar tests and INRs and explains about normal clotting time and why it's important to know what the INR is of somebody who's on warfarin. And that warfarin can be really tricky. There's lots of things that it interacts with and (he) explains some of the interactions. He says it can be dangerous if the patient’s clotting time is too long but that it can be reversed with vitamin K. He explains how vitamin K works and then he talks about the interaction between pharmacy and general practice and how a GP might be happy for pharmacists to make changes to warfarin doses through standing orders. The social work student [shadower] affirms saying “OK, OK I didn’t know about this”. (FN SW student shadowing a Pharm student)

Students self-reported the impact of the confident personality style on keeping the collaborative work going.

I find in a group like that you've kind of got one that might be more like (the) decision maker or …another one might keep the spirits a bit high if we're lacking motivation or crack a few jokes or whatever. …. Yes, still all of us, all did work and research, and pulled that together. (FG OT student)

## Discussion

Students shadowing registered health professionals from another discipline has been accepted as an effective IP learning activity and are included in some IPE curricula. The present study used a social constructivist approach (acknowledging the active role of learners, and social aspects of learning)^18^ to further demonstrate that multifaceted IP learning occurs when students of different disciplines engage in peer shadowing. Importantly, our study reveals *what* and *how* IP students learn from each other when engaging in peer shadowing, but also that other aspects influence learning (which are not necessarily obvious).

Similar to Rotz et al’s^11^ peer shadowing study, our study found students learned about roles and responsibilities, skills and attitudes, team performance, team relationships and collaborative team care - these being recognized IP competencies. However, by using CSOR sequenced methods whereby independently collected observation data are prioritised ahead of self-report data in both data collection and analysis, our study extends Rotz et al’s previous findings,^11^ providing a richer understanding of *how* students acquire these IP competencies through peer shadowing, and that this learning could be reciprocal or bi-directional. Students also learned about professional training programmes, professional behaviours and ethics, and social relationships.

Both shadowee and shadower students in our study were observed to take the IPE learning activity seriously and some talked about being anxious or nervous before the activity started, seemingly because they felt responsible for their fellow student’s learning. It was clear most shadowee students had thought about what to talk about or demonstrate in advance of the shadowing activity (i.e. they prepared for the peer teaching). Being socially connected through living together and therefore being comfortable to explain and do things together seems to have enhanced the shadowee student’s ability to teach, and many examples of sophisticated peer-teaching practice were revealed. Peer teaching is said to be active teaching of peers by students who are at the same level cognitively and socially^29^ with one study describing how to prepare peer teachers.^30^ In our study, shadowee students were not instructed to be peer teachers, however they appeared to naturally use a range of educational approaches, noting *when* and *what* to observe and affirmatively checking understanding. These behaviours, and peer teaching per se, are known to create safe learning environments which respect, support and empower students to ask any sorts of questions and practice skills, fostering ‘deep’ rather than superficial learning,^31^ without fear of being criticised.^32^ This is particularly important for IP learning where the impact of the hidden curriculum may mean some students have internalised views of the disciplinary hierarchy and may feel anxious about working with particular disciplines,^33^ or may have had previous negative experiences in IP learning.^34^ In this study as in others, students commented on the intrinsic reward of teaching a peer in the shadowing learning activity.^35,36^ Peer teaching which intentionally aims for bi-directional and reciprocal learning has been previously described in uni-professional learning^37^ but appears to be less common in IPE, and when undertaken, has involved peer teachers a year or more senior.^35,36^ This study shows students of an equivalent level of learning (final-year students in this case) can undertake successful peer teaching roles.

While we observed numerous positive examples of active peer-teaching and active learning from students during peer shadowing, we did note a lack of student contribution/peer-teaching during one of the examples. This may have been due to personal factors such as social anxiety, perceived hierarchical differences, general lack of confidence or feeling nervous or intimidated by a tutor, if present.^38^ Nonetheless, even in this case, the shadowing student still reported a positive learning experience in an electronic discussion board post.

Shadowing students also appeared to learn through being situationally aware, meaning they deliberately noticed what was happening around them, listening to conversations often involving or between other disciplines, watching the actions of colleagues and/or noting wider aspects of care and context.^13^ Although situational awareness is written about in relation to simulation IPE (usually regarding patient-safety topics^39^), this aspect does not seem to have been reported in IPE delivered in routine workplace settings. Utilising situational awareness may be a result of the students in this study being final-year students and having a greater readiness to actively seek out new knowledge and to be interprofessionally curious. Alternatively it could be as a result of the greater psychological safety created in the shadowing learning activity, enabling them to actively engage in another discipline’s world.^40,41^ Irrespective, this ability to learn more broadly from other disciplines is believed to be a critical skill for engaging in effective teamwork^42^ and could foster an IP identity (rather than a uni-disciplinary identity) which is believed to be an important aspect of building interprofessionalism.^43^ Viewed through the social constructivist lens,^18^ students’ IP learning occurred through experiential actions (learning by doing), social interactions and reflections. These actions, interactions and reflections occurred in clinical, non-clinical and social locations and were facilitated using approaches such as encouragement, empathy and humour, boosted in some situations by individual students’ enthusiasm and confidence. Tutors could also positively support learning. Importantly, all IP learning observed during shadowing appeared to have been actively influenced by existing social arrangements between students. Recalling that over the course of the five weeks, students lived together in shared accommodation and undertook other interactive IPE learning activities, it appears that by the time students engaged in the shadowing activities (weeks 2 onwards), they had already socially bonded, through sharing household activities and undertaking weekend excursions in a largely unknown remote rural area.^44^

### Strengths, Limitations and Recommendations

A strength in this study was the CSOR emphasis on the detailed ethnographic observations of a small number of examples which revealed a deep understanding of what actually happens and *how* students learn during peer-shadowing. Detailed field notes captured the non-verbal behaviours such as nods or other gestures which signaled assent or agreement^45,46^ as well as the minimal verbal encouragers and affirmations.^47^ Much of this would not have emerged using only self-reported accounts, however the participants’ perceptions were also important as this study showed that not all learning can be captured by observation. Learning that was not directly observable was instead evident in the written posts on electronic discussion boards and weekly surveys conducted throughout our study.

A potential limitation of this study is the shadowing learning activity occurs in an extended length IPE programme which has a particular curriculum design including students living in shared accommodation. The programme deliberately adopts a social constructivist approach^18^ ensuring activities are experiential and include reflection and knowledge sharing and social interaction occurs right from when the students move into the accommodation. Further research may be needed to see if the same learning outcomes from peer shadowing can be achieved in a shorter time frame and between students who do not already know each other socially. A second possible limitation is that most students self-selected to join the 5-week IPE programme, making these participants more likely to be positively predisposed to interprofessionalism. Being senior and in their final year on the cusp of graduating, they could have been more motivated than junior students to gain as much as they could from this interprofessional learning activity. Finally, the isolated rural location could have meant students responded by relying on each other for social support.

For educators wishing to introduce peer-shadowing into IPE curricula where students are not together for an extended length of time, it is possible a greater degree of preparation or briefing is needed before peer shadowing activities to achieve the learning outcomes we have observed. Ideally the shadowee student might be told in advance to think about what is interesting and unique about their discipline and how this could be best discussed or demonstrated, giving due consideration if patients are involved. The shadower student could be advised to list questions they always wanted to know about the other discipline and not be hesitant to ask them. A template with prompts of what to observe may also be helpful and used in reflection.^15^ Moreover, it seems wise to set out ground rules in advance about what questions or requests can be made in front of patients or staff.

Peer shadowing could also be set up as a two-part activity where student pairs could shadow each other in reverse, which was not done in this study.

We consider peer shadowing is best undertaken with senior or final-year students; they are more likely to be capable and confident to explain their discipline, use effective educational techniques and competently demonstrate and/or teach some of their discipline’s skills.

Preparation for supervisors or other staff to support peer shadowing (even if they are not always present with students) should also be given consideration. This includes knowing the aims of peer shadowing and preparing examples to share of times when they have drawn on another discipline’s knowledge or advice. As observed in our study, tutors can prompt students of another discipline with examples of their skill set when it is apparent that the student may not realise they have something to contribute. The latter would support bi-directional or reciprocal learning. As shown with other interprofessional learning activities, following up the shadowing learning activity with some form of reflective activity has the potential to consolidate learning.^38,48^ In our study students frequently and spontaneously reflected on shadowing during end-of-week debriefs, and through an electronic discussion board.

## Conclusions

This study shows peer shadowing is a form of shadowing in its own right. Shadowing can be defined as a learning activity which gives a student of one discipline (shadower) a chance to observe in real-time and usually in a workplace setting, the clinical practice of a student of another discipline (shadowee), where that student knows they are being observed. As part of an IPE curriculum where interaction and social engagement also occur, peer shadowing between final-year students supports comprehensive, meaningful learning about interprofessional collaboration as well as other areas of clinical and non-clinical practice, and professional and social behaviours. Academic staff involved in peer shadowing should prebrief the students (both shadowee and shadower) about the goals of the learning activity and advise them to prepare in advance by thinking about what they could demonstrate (shadowee) and what they could learn (shadower). When possible, clinical tutors who are involved in supervising peer shadowing should actively support the activity by providing examples from their experience that illustrate the skill sets both disciplines.

## Supplemental Material

Supplemental Material - Student Learning in an Interprofessional Peer Shadowing Activity Embedded in an IPE Curriculum: An Aotearoa New Zealand Ethnographic Case Study Supplemental Material for Student Learning in an Interprofessional Peer Shadowing Activity Embedded in an IPE Curriculum: An Aotearoa New Zealand Ethnographic Case Study by Eileen McKinlay, Julia Myers, Linda Gulliver and Sonya Morgan in Journal of Medical Education and Curricular Development.

## Data Availability

The research dataset is not available.*
