# Supplementary material for: Student Learning in an Interprofessional Peer Shadowing Activity Embedded in an IPE Curriculum: An Aotearoa New Zealand Ethnographic Case Study
Source: J Med Educ Curric Dev. 2026 May 3;13:23821205261448297. doi: 10.1177/23821205261448297 (PMC13157537; doi:10.1177/23821205261448297)
Supplement: Supplemental Material - Student Learning in an Interprofessional Peer Shadowing Activity Embedded in an IPE Curriculum: An Aotearoa New Zealand Ethnographic Case Study [file sj-pdf-1-mde-10.1177_23821205261448297.pdf]

Supplementary

OBSERVATION RECORDING TEMPLATE

Aim to observe student interaction before, during and after the scheduled interprofessional activity. Prioritise observing student interaction (including pre-briefing, debriefing)

1. CONTEXTUAL DETAILS (used to categorise for cultural-discursive, material-economic, and social-political elements)

Date:	Time:
Site:	Observer:
Actors present (note discipline):	

Summary of activities observed:

Description of context (including artefacts, posts, resources, set up of rooms):
[Note: take photos to show the location of actors in the room where possible. Consider a diagram]

Description of the mood/tone of the room (emanating from students):

2. UNSTRUCTURED FIELD NOTES: Write down as much of the content of the interaction/interprofessional learning between students as possible in chronological order. Add time stamps, and note if nothing happens (this will be used to categorise for sayings, doings, relatings). ***[NB: where sayings seem pertinent, note the sayings verbatim if possible]***

NOTE: write or dictate your reflections separately
